# Lipidomic profiling of exosomes from colorectal cancer cells and patients reveals potential biomarkers

**DOI:** 10.1002/1878-0261.13223

**Published:** 2022-06-14

**Authors:** Mohammed I. Y. Elmallah, Pablo Ortega‐Deballon, Laure Hermite, Jean‐Paul Pais‐De‐Barros, Jessica Gobbo, Carmen Garrido

**Affiliations:** ^1^ INSERM UMR 1231 Laboratoire d’Excellence LipSTIC and « Equipe labéllisée par la Ligue Nationale contre le Cancer » Dijon France; ^2^ Faculty of Medicine and Pharmacy Université de Bourgogne Franche‐Comté Dijon France; ^3^ Chemistry Department Faculty of Science Helwan University Cairo Egypt; ^4^ Centre Hospitalier Universitaire Dijon France; ^5^ Anti‐cancer Center Georges‐François Leclerc Dijon France; ^6^ Lipidomic Platform Dijon France; ^7^ Clinical investigation center INSERM 1432 CHU Dijon‐Bourgogne France

**Keywords:** biomarkers, colorectal cancer, exosomes, lipidome, mass spectroscopy

## Abstract

Strong evidence suggests that differences in the molecular composition of lipids in exosomes depend on the cell type and has an influence on cancer initiation and progression. Here, we analyzed by liquid chromatography–mass spectrometry (LC‐MS) the lipidomic signature of exosomes derived from the human cell lines normal colon mucosa (NCM460D), and colorectal cancer (CRC) nonmetastatic (HCT116) and metastatic (SW620), and exosomes isolated from the plasma of nonmetastatic and metastatic CRC patients and healthy donors. Analysis of this exhaustive lipid study highlighted changes in some molecular species that were found in the cell lines and confirmed in the patients. For example, exosomes from primary cancer patients and nonmetastatic cells compared with healthy donors and control cells displayed a common marked increase in phosphatidylcholine (PC) 34 : 1, phosphatidylethanolamine (PE) 36 : 2, sphingomyelin (SM) d18 : 1/16 : 0, hexosylceramide (HexCer) d18 : 1/24 : 0 and HexCer d18 : 1/24 : 1. Interestingly, these same lipids species were decreased in the metastatic cell line and patients. Further, levels of PE 34 : 2, PE 36 : 2, and phosphorylated PE p16 : 0/20 : 4 were also significantly decreased in metastatic conditions when compared to the nonmetastatic counterparts. The only molecule species found markedly increased in metastatic conditions (in both patients and cells) when compared to controls was ceramide (Cer) d18 : 1/24 : 1. These decreases in lipid species in the extracellular vesicles might reflect function‐associated changes in the metastatic cell membrane. Although these potential biomarkers need to be validated in a larger cohort, they provide new insight toward the use of clusters of lipid biomarkers rather than a single molecule for the diagnosis of different stages of CRC.

## Graphical abstract

Lipid dysregulation‐induced structural changes of the plasma membrane in cancer modulate cellular homeostasis, signaling pathways, and can affect colorectal cancer (CRC) initiation and progression. Since these changes are also found in the membrane lipid bilayer of the tumor‐released exosomes, mass spectroscopy can help to define an exosomal lipidomic signature to find noninvasive circulant diagnostic/prognostic biomarkers for different CRC stages.

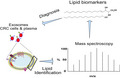

**Abbreviations**
Alix: ALG‐2‐interacting protein X
C: cholesterol
Cer: ceramide
CRC: colorectal cancer
ER: endoplasmic reticulum
ESI: electrospray ionization
GC‐MS: gas chromatography‐mass spectroscopy
HexCer: hexosylceramide
LC‐MS: liquid chromatography–mass spectrometry
LC‐MS/MS: liquid chromatography mass spectroscopy/mass spectroscopy
NTA: nanosight tracking analysis
PC: phosphatidylcholine
PE: phosphatidylethanolamine
PI: phosphatidylinositol
pPC: phosphorylated phosphatidylcholine
pPE: plasmalogen
PS: phosphatidylserine
SM: sphingomyelin
Tsg101: tumor susceptibility gene 101 protein

## 1 Introduction

Exosomes, extracellular nanovesicles (50–200 nm in diameter) of endosomal origin secreted by living cells into the extracellular environment [1], harbor a bioactive cargo of proteins, nucleic acids, and lipids [2]. These molecules can be transported by exosomes to different cell targets influencing their phenotype and physiological behavior. Tumor‐derived exosomes have been reported to play a major role in cancer initiation and progression for instance in colorectal cancer (CRC) [3].

Dysregulation of lipid metabolism can affect cellular homeostasis and signaling pathways, which subsequently influences the process of cell proliferation and differentiation. Such change in the dynamic structure of the plasma membrane lipid bilayer has a major contribution to the onset of various diseases including cancer [4]. The majority of exosomal lipids are mainly localized in the membrane and have been reported to play a role in the biogenesis, secretion, fusion, and uptake of exosomes [5]. Although the molecular composition of lipids in exosomes depends on the cell type, it has been found that the membrane of the exosomes compared to that of the cell from which they originate is enriched in cholesterol (C), sphingomyelin (SM), glycosphingolipids, and glycerophospholipids [6].

Exploration of exosomal lipids as noninvasive circulant cancer biomarkers has only recently started. So far, just a few studies have analyzed the lipidomic profile of exosomes derived from breast [3], ovarian [7], and prostate [1] cancer cell lines. For CRC, only the lipid composition analysis of exosome‐derived from the colorectal cancer LIM1215 cell line by mass spectrometry has been reported [8]. Therefore, further lipidomic analysis in colorectal cancer cells‐derived exosomes is needed to understand in‐depth the role of exosomes in cancer initiation and progression and to identify specific diagnostic/prognostic lipid biomarkers for different stages of CRC. In this pilot study, we analyzed the lipidomic signature of exosomes derived from CRC cell lines and patients by LC‐MS. The results revealed that exosomes from both nonmetastatic and metastatic cell lines and those from the plasma of patients displayed similar significant variations in the lipidomic signature of certain lipid molecular species, particularly in glycerophospholipids and sphingolipids compared with their corresponding controls.

## 2 Materials and methods

### 2.1 Cell lines and patients

The normal colonic epithelial cell line NCM460D (RRID:CVCL_IS47) was purchased from In Cell (San Antonio, TX, USA). HCT116 (RRID:CVCL_0291) and SW620 (RRID:CVCL_0547) cell lines were purchased from American Type Culture Collection (Manassas, VA, USA). All experiments were performed with cell‐free mycoplasma using a mycoplasma detection kit (MycoAlert, Lonza Pharma&Biotech, Basel, Switzerland). Cell lines were grown in Dulbecco′s Modified‐Eagle′s Medium (DMEM) supplemented with 10% FBS. The patient’s blood samples were obtained from the University Hospital of Dijon (France). The study was conducted in accordance with the Declaration of Helsinki with an approved written consent form for each patient (CPP ESTI: 2014/39; N°ID: 2014‐A00968‐39). This study was approved by the local ethics committee (IRB 00010311).

### 2.2 Isolation of exosomes

Cells were cultured in DMEM supplemented with 10% FBS (exosome depleted) until reached 80% confluence. Exosomes derived from this conditioned medium and from the plasma of patients were performed by differential ultracentrifugation and filtration as previously described [9]. The concentration and size distribution of exosomes were measured by Nanoparticle tracking analysis (NanoSight NS300, Malvern, UK) and stored at −80 °C until use.

### 2.3 Western blot analysis

Twenty µg of proteins from the exosome lysates (Bradford assay) were loaded on 10% SDS‐PAGE followed by blotting the separated proteins onto a polyvinylidene difluoride (PVDF) membrane (Amersham GE Healthcare Life Sciences, Bukinghamshire, UK). The membrane was incubated with the appropriate primary antibodies including mouse anti‐ ALG‐2‐interacting protein X (Alix) mAb‐3A9 (Fisher Scientific, Waltham, MA, USA), mouse anti‐tumor susceptibility gene 101 protein (Tsg101) mAb‐7964 (C‐2), mouse anti‐syntenin‐1 mAb‐10036 (S‐31) and mouse anti‐CD9 mAb‐1318 (C‐4) from Santa Cruz Biotechnology, Heidelberg, Germany, mouse anti‐CD63 mAb‐NBP2‐42225SS (Novus Biologicals, Centennial, CO, USA), rabbit anti‐calnexin mAb‐2679 (C5C9) from Cell Signaling Technology (Leiden, Netherland), and mouse anti‐β‐actin mAb‐A5441 (Sigma Aldrich, St Louis, MO, USA) overnight at 4 °C. Following the washing steps, the membrane was incubated with the corresponding secondary antibodies. Proteins were detected using an enhanced chemiluminescence ECL‐kit (Advansta, San Jose, CA, USA).

### 2.4 Lipid extraction

LC‐MS/MS quality grade chemicals were from Sigma Aldrich (Saint‐Quentin Fallavier, France) and solvents were purchased from Fischer Scientific (Illkirch, France). Lipids were extracted according to the method of Bligh and Dyer as previously described [10].

### 2.5 Targeted lipidomics

Phospholipids and ceramides were analyzed on a 1200 6460‐QqQ LC‐MS/MS system equipped with an Electrospray ionization (ESI) source (Agilent Technologies) as previously described [10]. Cholesterol was measured by gas chromatography–mass spectroscopy (GC‐MS) using 10 or 15 µL of the Bligh and Dyer extracts obtained from plasma or cellular exosomes, respectively [11].

### 2.6 Statistics

Lipid species were normalized to total cholesterol and analyzed by Two‐way ANOVA followed by Tukey’s multiple comparisons test. Data were considered statistically significant when *P* values ≤ 0.05. The statistical analysis was performed using graphpad prism version 8.0.0 for Windows, GraphPad Software (San Diego, CA, USA).

## 3 Results and Discussion

Analysis of exosomes derived from normal colon mucosa NCM460, nonmetastatic HCT116 and metastatic SW620 CRC cells by nanosight tracking analysis (NTA) did not reveal any significant differences in their average size. However, NCM460 and HCT116 showed a higher average concentration of exosomes compared with SW620 (Fig. S1A). Concerning NTA analysis of plasma‐derived exosomes (*n* = 12) of nonmetastatic, metastatic CRC patients, and healthy donors, no significant differences were found among the three groups in both size and concentration (Fig. S2A). Western blot analysis showed that the isolated nanovesicles from all cells (Fig. S1B) and patients (Fig. S2B) were positive for the exosomal marker proteins Tsg101, Alix, syntenin‐1, CD9, and CD63 while negative for the endoplasmic reticulum (ER) marker calnexin.

The lipidomic profile of exosomes, analyzed by LC‐MS and normalized to total cholesterol (nm) lead to the quantification of 175 lipid species in exosomes from both NCM460 and HCT116, 132 lipid species in SW620, and 178 lipid species in the three groups of plasma‐derived exosomes (healthy donors, nonmetastatic, and metastatic, Table S1). The relative distribution of lipid compositions was considerably different among the exosomes. However, all exosomes were relatively abundant in sphingolipids (Figs S1C and S2C) and PC (Figs S1D and S2D), which is in agreement with the hypothesis that exosomal membranes harbor lipid raft‐like domains [12] and are enriched in PC subclasses [8, 13]. In metastatic patients, like metastatic SW620, exosomes possessed a smaller mole ratio of PS compared with nonmetastatic patients (Fig. S2D). Cholesterol was chosen to normalize the lipidomic, as it was an abundant lipid in all samples and no significant differences were detected among the different exosomes in the mole ratio of cholesterol (Fig. S3).

**Fig. 1:**
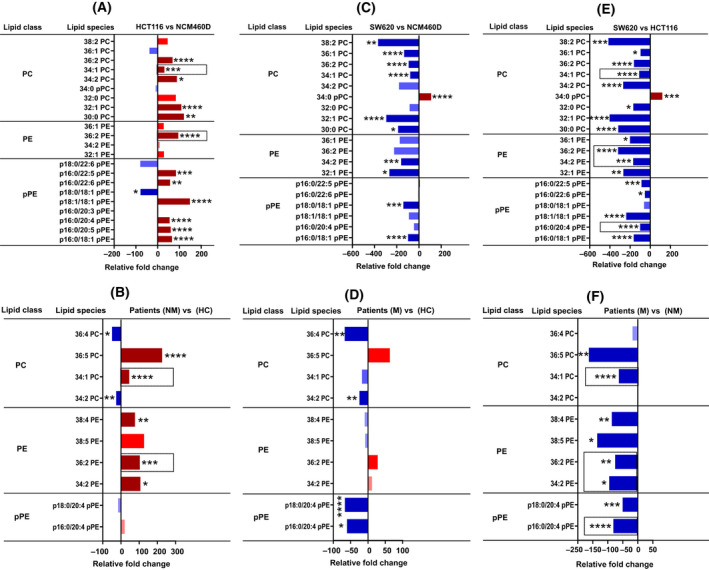
Lipidomic analysis of phosphatidylcholine (PC), phosphatidylethanolamine (PE), and plasmalogen (pPE) content in CRC cell lines and patients‐derived exosomes. Lipidomic analysis of phospholipid subclasses, phosphatidylcholine (PC), phosphatidylethanolamine (PE), and plasmalogen (pPE) individual species in exosomes derived from cell lines (NCM460D, HCT116, and SW620), and from metastatic, nonmetastatic patients, and healthy donors (*n* = 4 for each group, pooled). Data are represented as the relative fold increase/decrease of the indicated individual lipid species normalized to total cholesterol. Shades of red represent increased values while shades of blue are for decreased values. Only the species found changed are shown. (A, B) Nonmetastatic vs control cells (A) and patients (B). (C, D) Metastatic vs control cells (C) and patients (D). (E, F) Nonmetastatic vs metastatic cells (E) and patients (F). Column bars surrounded by a dashed line indicate the common lipid species that were found in cell lines and were confirmed in patient‐derived exosomes. Data were analyzed by two‐way ANOVA followed by the Tukey’s multiple comparison test. Significant *P* values of four independent replicates were *P ≤ 0.05, **P ≤ 0.01, ***P ≤ 0.001, ****P ≤ 0.0001, *n* = 4.

**Fig. 2:**
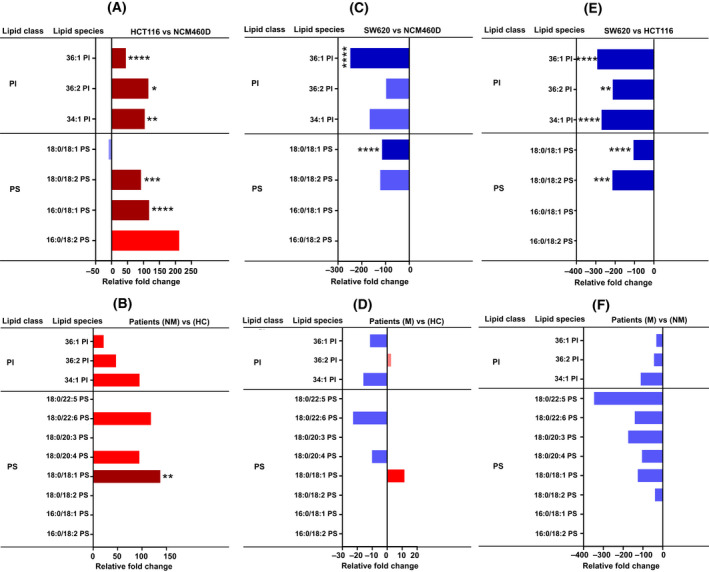
Lipidomic analysis of phosphatidylinositol (PI) and phosphatidylserine (PS) content. Analysis of phospholipid subclasses, phosphatidylinositol (PI), and phosphatidylserine (PS) molecular species in metastatic and nonmetastatic exosomes (cell lines and patients, *n* = 4 for each group, pooled and the same number of healthy donors) represented in relative fold change percentage normalized to total cholesterol. Shades of red represent increased values while shades of blue are for decreased values. Only the species found changed are shown. (A, B) Nonmetastatic vs control cells (A) and patients (B). (C, D) Metastatic vs control cells (C) and patients (D). (E, F) Metastatic vs nonmetastatic cells (E) and patients (F). Data were analyzed by two‐way ANOVA followed by the Tukey’s multiple comparison test. Significant *P* values of four independent replicates were *P ≤ 0.05, **P ≤ 0.01, ***P ≤ 0.001, ****P ≤ 0.0001, *n* = 4.

**Fig. 3:**
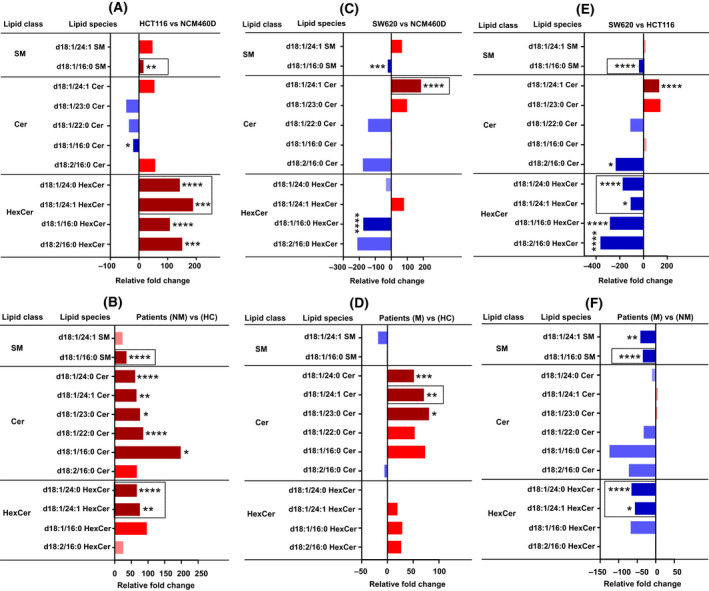
Lipidomic analysis of sphingolipid content. Analysis of sphingolipid subclasses, sphingomyelin (SM), and ceramide (Cer) molecular species in metastatic and nonmetastatic exosomes (cell lines and patients, *n* = 4 for each group, pooled, and the same number of healthy donors) represented in relative fold change percentage normalized to total cholesterol. Shades of red represent increased values while shades of blue are for decreased values. Only the species found changed are shown. (A, B) Nonmetastatic vs control cells (A) and patients (B). (C, D) Metastatic vs control cells (C) and patients (D). (E, F) Metastatic vs nonmetastatic cells (E) and patients (F). Column bars surrounded by a dashed line indicate the common lipid species that were found in cell lines and were confirmed in patient‐derived exosomes. Data were analyzed by two‐way ANOVA followed by the Tukey’s multiple comparison test. Significant *P* values of four independent replicates were *P ≤ 0.05, **P ≤ 0.01, ***P ≤ 0.001, ****P ≤ 0.0001, *n* = 4.

The lipidomic analysis was next extended to the individual molecular species of the identified lipid subclasses. Figures 1, 2, 3 show the subclasses for which differences were obtained when comparing controls with cancer and/or nonmetastatic from metastatic conditions (raw data obtained for all subclasses analyzed are shown in Figs [Link], [Link], [Link], [Link], [Link]). Considering PC subclass, the molecular species, PC 30:0, 32:1, 34:2, 34:1, and 36:2 were significantly increased in HCT116 compared with control NCM460 (Fig. 1A). Interestingly, all these PC species were decreased in SW620 along with PC 32:0, 36:1, and 38:2 when compared both with the control and the nonmetastatic HCT116 (Fig. 1C,E). Only the molecular species phosphorylated PC 34:0 (pPC 34:0) was markedly increased in SW620 (Fig. 1C,E). In accord with this result, an increased level of PC molecular species 32:1 was reported in CRC tissues [14]. For CRC plasma‐derived exosomes, nonmetastatic patients revealed a significant enrichment in the PC 34:1 and 36:5 molecular species compared with the healthy controls and metastatic patients (Fig. 1B,F). Moreover, exosomes derived from cancer patients, compared with the healthy donor, showed a decrease in the level of PC 34:2 and 36:4 individual species (Fig. 1B,D). Interestingly, the significant increase in the PC molecular species 34:1 in nonmetastatic HCT116‐exosomes was also observed in exosomes derived from plasma of nonmetastatic CRC patients when compared with their corresponding normal counterparts (Fig. 1A,B). It should be noted that the level of PC 34:1 was also found to be increased in the exosomes derived from NB26 and PC‐3 prostate cancer cell lines [1].

For the PE subclass, the molecular species PE 32:1, 34:2, 36:2, and 36:1 were significantly decreased in SW620 compared with NCM460 and HCT116 (Fig. 1C,E). Like the nonmetastatic HCT116‐exosomes, plasma exosomes derived from nonmetastatic patients revealed a significant increase in the PE individual species 36:2, compared with the control and metastatic patients (Fig. 1A,B). Similarly, exosomes derived from metastatic both SW620 cells and patients displayed a significant decrease in the level of PE 34:2 and 36:2 molecular species compared with their nonmetastatic counterparts (Fig. 1E,F). In addition, PE 38:5 and 38:4 were also found decreased in exosomes from metastatic patients (compared with healthy donors and nonmetastatic patients; Fig. 1D,F).

Cancer cells have been reported to be characterized by increased levels of ether‐linked lipids like plasmalogen (pPE). This finding evoked several research groups to investigate ether‐linked lipids as potential diagnostic biomarkers in cancer [15]. The molecular mechanism by which plasmalogen triggers cancer cell proliferation was suggested to be due to the activation of phosphatidylinositol 3‐kinase [16]. Another study reported a decrease in the level of ether‐linked lipids in the serum of pancreatic and esophageal cancer patients [17]. Here, we found that six pPE molecular species, p16:0/18:1, p16:0/20:5, p16:0/20:4, p18:1/18:1, p16:0/22:6, and p16:0/22:5, were significantly increased in HCT116‐exosomes when compared with NCM460 (Fig. 1A,1E). These results may suggest a higher membrane fluidity of HCT116‐exosomes due to the significant incorporation of plasmalogen, which may facilitate the capture of oncogenic factors [18]. An increase in the pPE molecular species p16:0 and 18:1 has already been reported in the CRC LIM1215 cell line and in CRC tissues, respectively [8, 19]. Concerning exosomes from metastatic patients, they displayed a marked decrease in p16:0/20:4 and p18:0/20:4 compared with healthy donors and nonmetastatic patients (Fig. 1D,F). Similarly, exosomes from both metastatic SW620 cells and patients displayed a marked decrease in the pPE molecular species p16:0/20:4 compared with their nonmetastatic counterparts (Fig. 1E,F). The decreased amount of plasmalogen in metastatic exosomes compared with the nonmetastatic might reflect different functions such as cancer cell detachment and dissemination.

HCT116‐exosomes showed an overall enrichment in the phosphatidylinositol (PI) molecular species with an obvious increase in PI 34:1, 36:2, and 36:1 compared with NCM460 and SW620 (Fig. 2A,C,E). In contrast, no significant change in PI individual species in plasma‐derived exosomes among the three groups was found (Fig. 2B,D,F).

The phosphatidylserine (PS) molecular species 16:0/18:1 and 18:0/18:2 were markedly increased in HCT116‐exosomes compared with NCM460 and SW620 (Fig. 2A,E). Moreover, PS 18:0/18:1 was significantly decreased in SW620‐exosomes compared with control and nonmetastatic HCT116‐exosomes (Fig. 2C,E). In contrast, no significant change in the level of PS was detected in all three groups of plasma‐derived exosomes (Fig. 2B,D,E). Only the molecular species PS 18:1/18:0 was significantly increased in nonmetastatic patients compared with the healthy donor (Fig. 2B).

Tumor‐derived exosomes were suggested to transport ceramide‐enriched lipid rafts to recipient cells thereby inducing oncogenic signaling pathways in the donor cell [20]. In this study, analysis of sphingolipids (SM and Cer) subclasses revealed that both nonmetastatic HCT116‐ and patient‐derived exosomes had a significant increase in SM d18:1/16:0, HexCer d18:1/24:1, and d18:1/24:0 (Fig. 3A,B). Interestingly, we found again that those same species were decreased in metastatic SW620‐exosomes and patients when compared to their corresponding controls (Fig. 3E,F). It is worth noting that HexCer d18:1/16:0 was reported to be increased in the plasma of patients with CRC, and was claimed as a potential biomarker for CRC [21]. The molecular species SM d18:1/16:0 was also found to be increased in exosomes derived from colon [8] and prostate [22] cancer cell lines. The nonmetastatic HCT116‐derived exosomes showed a significant enrichment in HexCer d18:1/24:1 and d18:1/24:0 molecular species (Fig. 3A). The same species were also found to be significantly higher in the exosomes from nonmetastatic patients along with Cer d18:1/16:0, d18:1/22:0, d18:1/23:0, d18:1/24:1, and d18:1/24:0 compared with healthy donors (Fig. 3B). The Cer d18:1/16:0, also known as C16 ceramide, has been reported to act as a lipid second messenger to regulate apoptosis and stress signaling [23]. Moreover, Cer d18:1/24:1 was markedly higher in metastatic SW620 and patients‐exosomes compared with their controls (Fig. 3C,D). This is in accord with a reported work demonstrating that Cer d18:1/24:1 was found to be enriched in the plasma of prostate cancer patients and PC‐3‐derived exosomes, which made the authors suggest a role for this ceramide in the progression of prostate cancer [1]. In addition, both metastatic and nonmetastatic patients compared with healthy controls showed a significant increase in Cer d18:1/23:0 and d18:1/24:0 (Fig. 3B,D).

## 4 Conclusion

In summary, targeted lipidomic analysis can enable the description of potential diagnostic/prognostic cancer biomarkers. Some signature profiling can already be proposed. For instance, markers when comparing controls and primary cancers might be PC 34:1, PE 36:2, SM d18:1/16:0, HexCer d18:1/24:0, and HexCer d18:1/24:1, and for the metastatic phenotype, we can propose the molecular species PE 34:2, PE 36:2, pPE 16:0/20:4, and Cer d18:1/24:1. Although more studies are needed to confirm these results and to unravel the role of each individual species in tumor lipid biology, our work opens the gate toward developing and designing a lipid signature for different disease states and toward understanding the role of exosomal lipids in signal transduction.

## Conflict of interest

The authors declare no conflict of interest.

## Author contributions

All authors shared an equal contribution to the preparation of the manuscript and the approval of the final version. MIY‐E performed all experiments and data analysis. PO‐D and LH provided the blood samples. JPP‐D contributed to targeted lipidomic analysis and data interpretation. JG and CG contributed to conceptualization, methodology, project administration, and funding acquisition.

### Peer Review

The peer review history for this article is available at https://publons.com/publon/10.1002/1878‐0261.13223.

## Supporting information


**Fig. S1.** Characterization and relative lipid compositions of exosomes from colon cancer and normal colon mucosa cells. (A) Average size (left panel) and concentration (right panel) of exosomes derived from normal colon mucosa NCM460D (black bars), nonmetastatic HCT116 (white bars), and metastatic SW620 (gray bars) CRC cell lines determined by nanosight tracking analysis (NTA). (B) Western blot analysis in the exosomes and cell lysates. Analyzed exosomes were positive for exosome protein markers including tumor susceptibility gene 101 protein (Tsg101), ALG‐2‐interacting protein X (Alix), syntenin‐1, and CD9. Calnexin was used as a negative control for exosomes, and β‐actin was used as a loading control. (C, D) Overall, lipid compositions (C) and mole percentage of lipid subclasses (D) in the exosomes from the indicated colon cell lines. Error bars represent the standard error mean (±SED) values of four independent replicates (*n* = 4). *P ≤ 0.05, **P ≤ 0.01, ***P ≤ 0.001, ****P ≤ 0.0001.

**Fig. S2.** Characterization and relative lipid compositions of exosomes from the plasma of colorectal cancer (CRC) patients (*n* = 8) and healthy donors (*n* = 4). (A) Average size (left panel) and concentration (right panel) of exosomes from healthy controls (HC‐black bars), nonmetastatic (NM‐white bars), and metastatic (M‐gray bars) CRC patients determined by nanosight tracking analysis (NTA). (B) Western blot analysis to identify the exosome protein markers including tumor susceptibility gene 101 protein (Tsg101), ALG‐2‐interacting protein X (Alix), CD63, and CD9. Calnexin was used as a negative control for exosomes. (C, D) Overall, lipid compositions (C) and mole percentage of lipid subclasses (D) in the depicted plasma‐derived exosomes. Error bars represent the standard error mean (±SED) values of four independent replicates (*n* = 4). *P ≤ 0.05, **P ≤ 0.01, ***P ≤ 0.001, ****P ≤ 0.0001.

**Fig. S3.** Determination of cholesterol by gas chromatography–mass spectroscopy (GC‐MS) in exosomes derived from both colorectal cancer (CRC) cell lines and patients compared with their corresponding controls (exosomes from NCM460 cells and healthy control, respectively). As depicted in the figure, no significant change in cholesterol was observed in the exosomes derived from both cell lines and patients. Data were analyzed by two‐way ANOVA followed by the Tukey’s multiple comparison test. Error bars represent the standard error mean (±SED) values of four independent replicates (*n* = 4). *P ≤ 0.05, **P ≤ 0.01, ***P ≤ 0.001, ****P ≤ 0.0001.

**Fig. S4.** Phosphatidylcholine (PC) species analysis normalized to total cholesterol of exosomes derived from (A) normal colon mucosa NCM460D, nonmetastatic HCT116, and metastatic SW620 colorectal cancer (CRC) cell lines and from (B) Plasma‐derived exosomes of healthy donors and CRC patients (nonmetastatic and metastatic) illustrating an overall enrichment of 34:1 PC molecular species in both nonmetastatic cells and patients compared with their corresponding controls and metastatic counterparts. Data were analyzed by two‐way ANOVA followed by the Tukey’s multiple comparison test. Error bars represent standard error mean values (±SEM, *n* = 4). *P ≤ 0.05, **P ≤ 0.01, ***P ≤ 0.001, ****P ≤ 0.0001.

**Fig. S5.** Phosphatidylethanolamine (PE) and plasmalogen (pPE) molecular species analysis normalized to total cholesterol in exosomes derived from (A) normal colon mucosa NCM460D, nonmetastatic HCT116 and metastatic SW620 colorectal cancer (CRC) cell lines and from (B) plasma‐derived exosomes of healthy donors and CRC patients nonmetastatic and metastatic (*n* = 4 for each group, pooled). Exosomes from both nonmetastatic HCT116 cells, and patients showed a significant increase of PE species 36:2 compared with their corresponding controls and metastatic counterparts. Metastatic SW620 cells and patients revealed a significant decrease in p16:0/20:4 pPE level compared with their nonmetastatic counterparts. Data were analyzed by two‐way ANOVA followed by the Tukey’s multiple comparison test. Error bars represent standard deviation (±SD, *n* = 4) values. *P ≤ 0.05, **P ≤ 0.01, ***P ≤ 0.001, ****P ≤ 0.0001.

**Fig. S6.** Phosphatidylinositol (PI) and phosphatidylserine (PS) molecular species analysis of exosomes derived from (A) normal colon mucosa NCM460D, nonmetastatic HCT116, and metastatic SW620 colorectal cancer (CRC) cell lines and from (B) plasma‐derived exosomes of healthy donors and CRC patients nonmetastatic and metastatic (*n* = 4 for each group, pooled) normalized to total cholesterol. HCT116‐derived exosomes are enriched in the PI molecular species PI 34:1, 36:2, and 36:1 compared with NCM460D and SW620. No significant change in the level of PI species was detected in all exosomes derived from the plasma of healthy donors and patients. Data were analyzed by two‐way ANOVA followed by the Tukey’s multiple comparison test. Error bars represent standard deviation (±SD, *n* = 4) values. *P ≤ 0.05, **P ≤ 0.01, ***P ≤ 0.001, ****P ≤ 0.0001.

**Fig. S7.** Analysis of sphingomyelin (SM) molecular species in exosomes derived from (A) normal colon mucosa NCM460D, nonmetastatic HCT116, and metastatic SW620 colorectal cancer (CRC) cell lines and from (B) plasma‐derived exosomes of healthy donors and CRC patients nonmetastatic and metastatic normalized to total cholesterol (*n* = 4 for each group, pooled). Both nonmetastatic HCT116‐ and patient‐derived exosomes revealed a marked increase in the level of d18:1/16:0 SM molecular species compared with their corresponding controls and metastatic counterparts. Data were analyzed by two‐way ANOVA followed by the Tukey’s multiple comparison test. Error bars represent standard deviation (±SD, *n* = 4) values. *P ≤ 0.05, **P ≤ 0.01, ***P ≤ 0.001, ****P ≤ 0.0001.

**Fig. S8.** Ceramide (Cer) molecular species analysis of exosomes derived from (A) normal colon mucosa NCM460D, nonmetastatic HCT116, and metastatic SW620 colorectal cancer (CRC) cell lines and from (B) plasma‐derived exosomes of healthy donors and CRC patients nonmetastatic and metastatic, normalized to total cholesterol (*n* = 4 for each group, pooled). Nonmetastatic HCT116‐ and patient‐derived exosomes had an increase in the level of hexosylceramide d18:1/24:1 HexCer and d18:1/24:0 HexCer molecular species compared with their controls and metastatic counterparts. Both metastatic SW620‐ and patient‐derived exosomes displayed a significant increase in the ceramide molecular species d18:1/24:1 compared with their controls. Data were analyzed by two‐way ANOVA followed by the Tukey’s multiple comparison test. Error bars ±SD, *n* = 4. *P ≤ 0.05, **P ≤ 0.01, ***P ≤ 0.001, ****P ≤ 0.0001.

**Table S1.** Total lipid ions quantified by liquid chromatography–mass spectrometry (LC‐MS) in exosomes derived from normal colon mucosa NCM460D, nonmetastatic HCT116, and metastatic SW620 colorectal cancer (CRC) cell lines and from plasma‐derived exosomes of healthy controls (HC), and CRC patients nonmetastatic (NM) and metastatic (M).

## Data Availability

All raw data that support the findings of this study are available from the corresponding author (cgarrido@u-bourgogne.fr) upon reasonable request.
