# Supplementary figures and images for: Lipidomic profiling of exosomes from colorectal cancer cells and patients reveals potential biomarkers
Source: Mol Oncol. 2022 Jun 14;16(14):2710–8. doi: 10.1002/1878-0261.13223 (PMC9298677; doi:10.1002/1878-0261.13223)

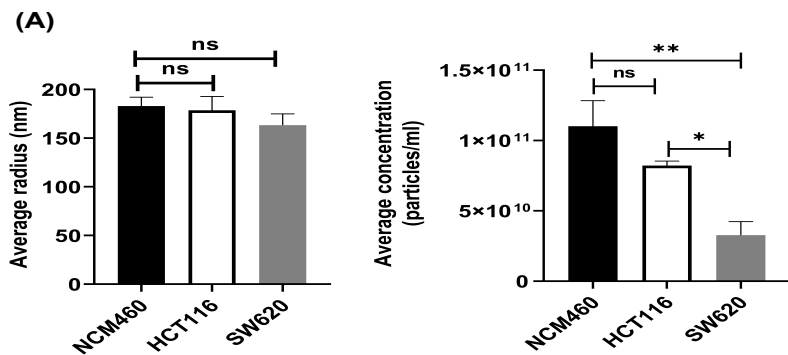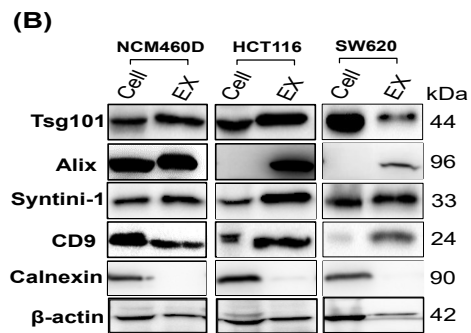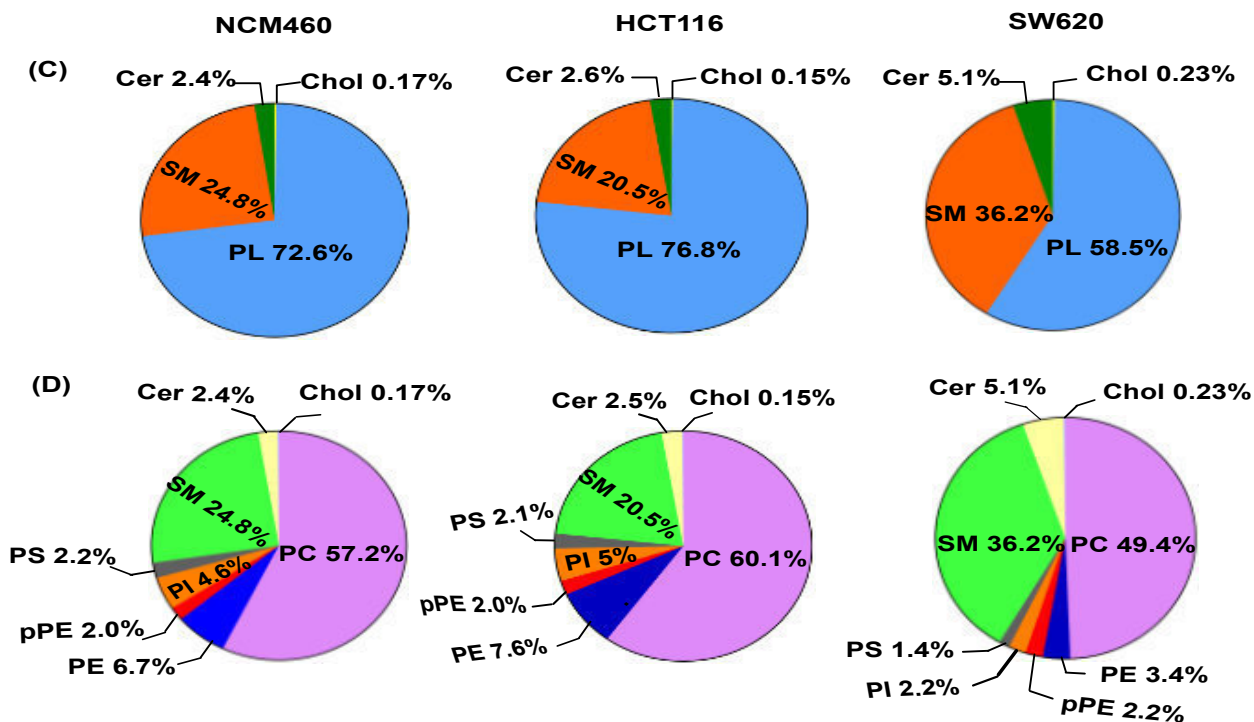

Supplement: Supplementary file 1 — Fig. S1. Characterization and relative lipid compositions of exosomes from colon cancer and normal colon mucosa cells. (A) Average size (left panel) and concentration (right panel) of exosomes derived from normal colon mucosa NCM460D (black bars), nonmetastatic HCT116 (white bars), and metastatic SW620 (gray bars) CRC cell lines determined by nanosight tracking analysis (NTA). (B) Western blot analysis in the exosomes and cell lysates. Analyzed exosomes were positive for exosome protein markers including tumor susceptibility gene 101 protein (Tsg101), ALG‐2‐interacting protein X (Alix), syntenin‐1, and CD9. Calnexin was used as a negative control for exosomes, and β‐actin was used as a loading control. (C, D) Overall, lipid compositions (C) and mole percentage of lipid subclasses (D) in the exosomes from the indicated colon cell lines. Error bars represent the standard error mean (±SED) values of four independent replicates (n = 4). *P ≤ 0.05, **P ≤ 0.01, ***P ≤ 0.001, ****P ≤ 0.0001. [file MOL2-16-2710-s004.pdf]

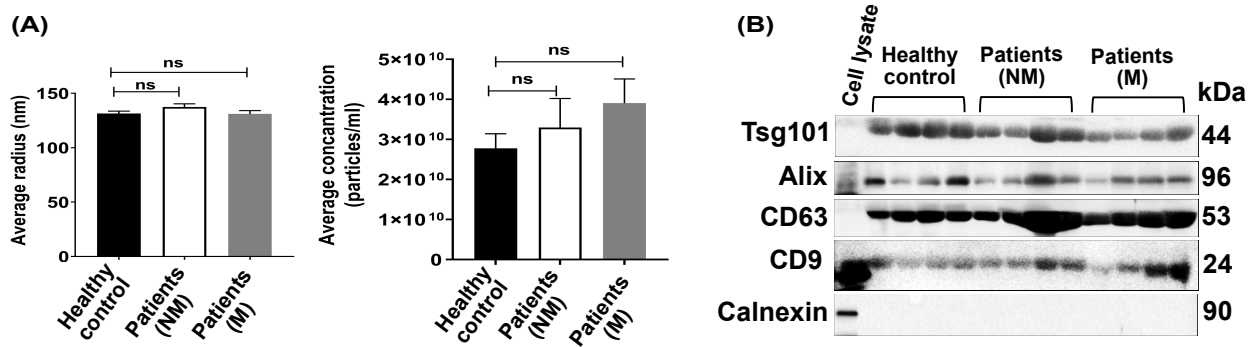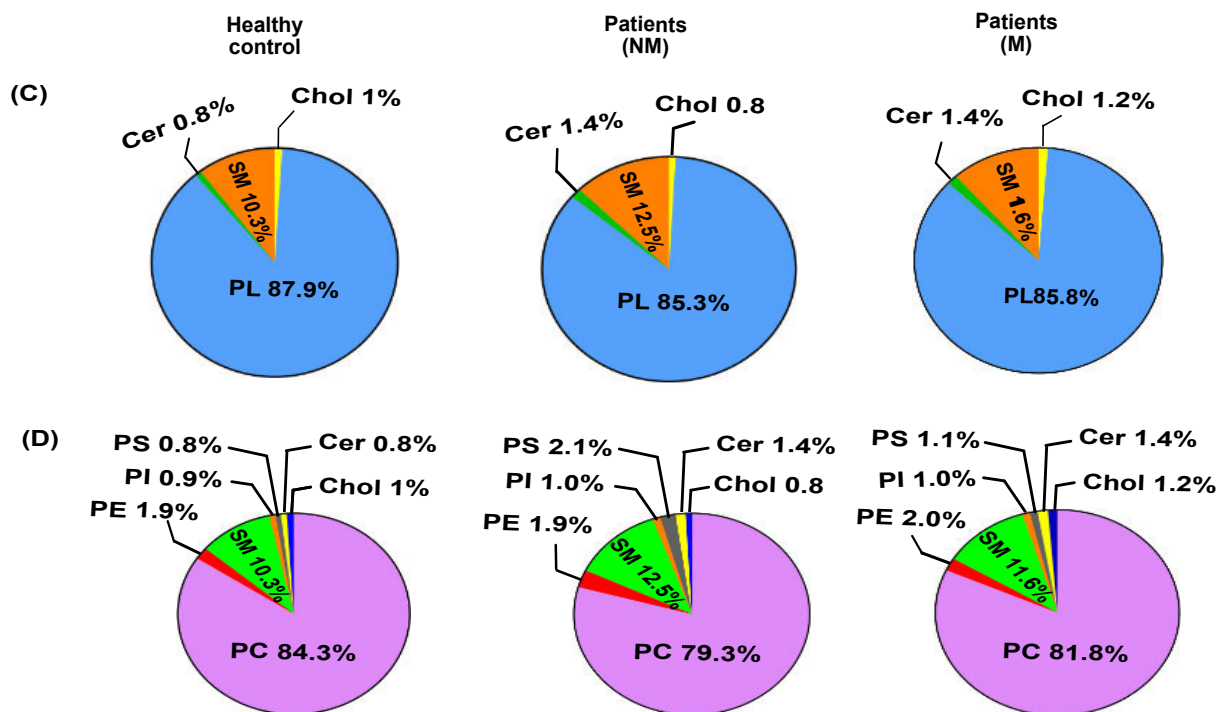

Supplement: Supplementary file 2 — Fig. S2. Characterization and relative lipid compositions of exosomes from the plasma of colorectal cancer (CRC) patients (n = 8) and healthy donors (n = 4). (A) Average size (left panel) and concentration (right panel) of exosomes from healthy controls (HC‐black bars), nonmetastatic (NM‐white bars), and metastatic (M‐gray bars) CRC patients determined by nanosight tracking analysis (NTA). (B) Western blot analysis to identify the exosome protein markers including tumor susceptibility gene 101 protein (Tsg101), ALG‐2‐interacting protein X (Alix), CD63, and CD9. Calnexin was used as a negative control for exosomes. (C, D) Overall, lipid compositions (C) and mole percentage of lipid subclasses (D) in the depicted plasma‐derived exosomes. Error bars represent the standard error mean (±SED) values of four independent replicates (n = 4). *P ≤ 0.05, **P ≤ 0.01, ***P ≤ 0.001, ****P ≤ 0.0001. [file MOL2-16-2710-s006.pdf]

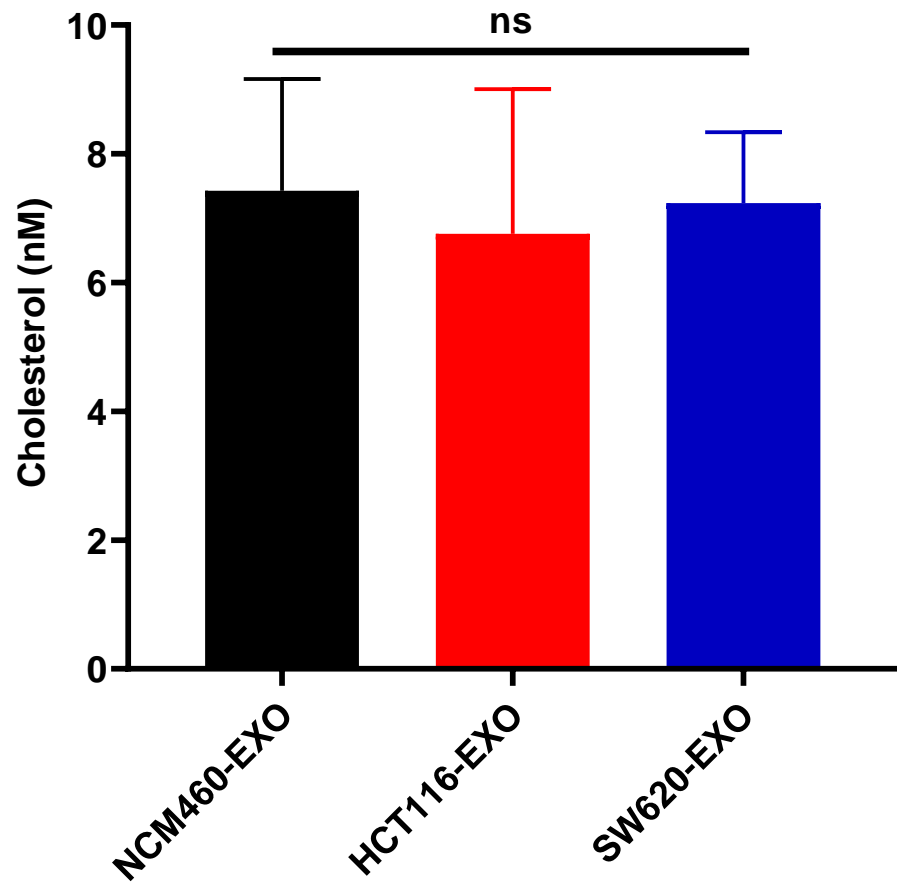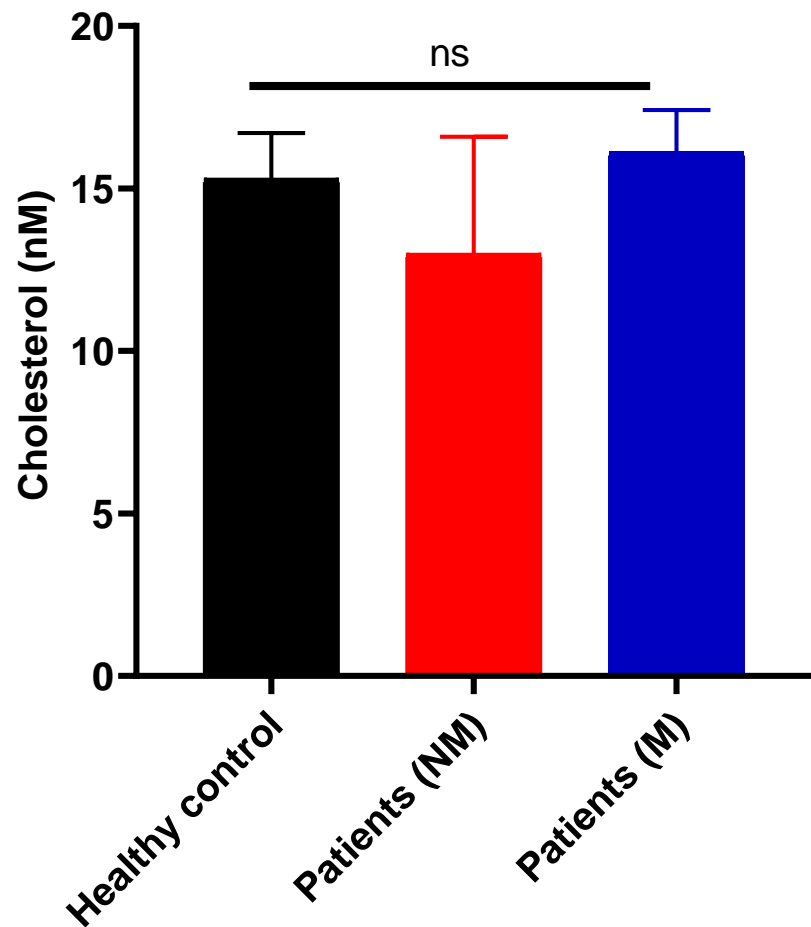

Supplement: Supplementary file 3 — Fig. S3. Determination of cholesterol by gas chromatography–mass spectroscopy (GC‐MS) in exosomes derived from both colorectal cancer (CRC) cell lines and patients compared with their corresponding controls (exosomes from NCM460 cells and healthy control, respectively). As depicted in the figure, no significant change in cholesterol was observed in the exosomes derived from both cell lines and patients. Data were analyzed by two‐way ANOVA followed by the Tukey’s multiple comparison test. Error bars represent the standard error mean (±SED) values of four independent replicates (n = 4). *P ≤ 0.05, **P ≤ 0.01, ***P ≤ 0.001, ****P ≤ 0.0001. [file MOL2-16-2710-s008.pdf]

(A)

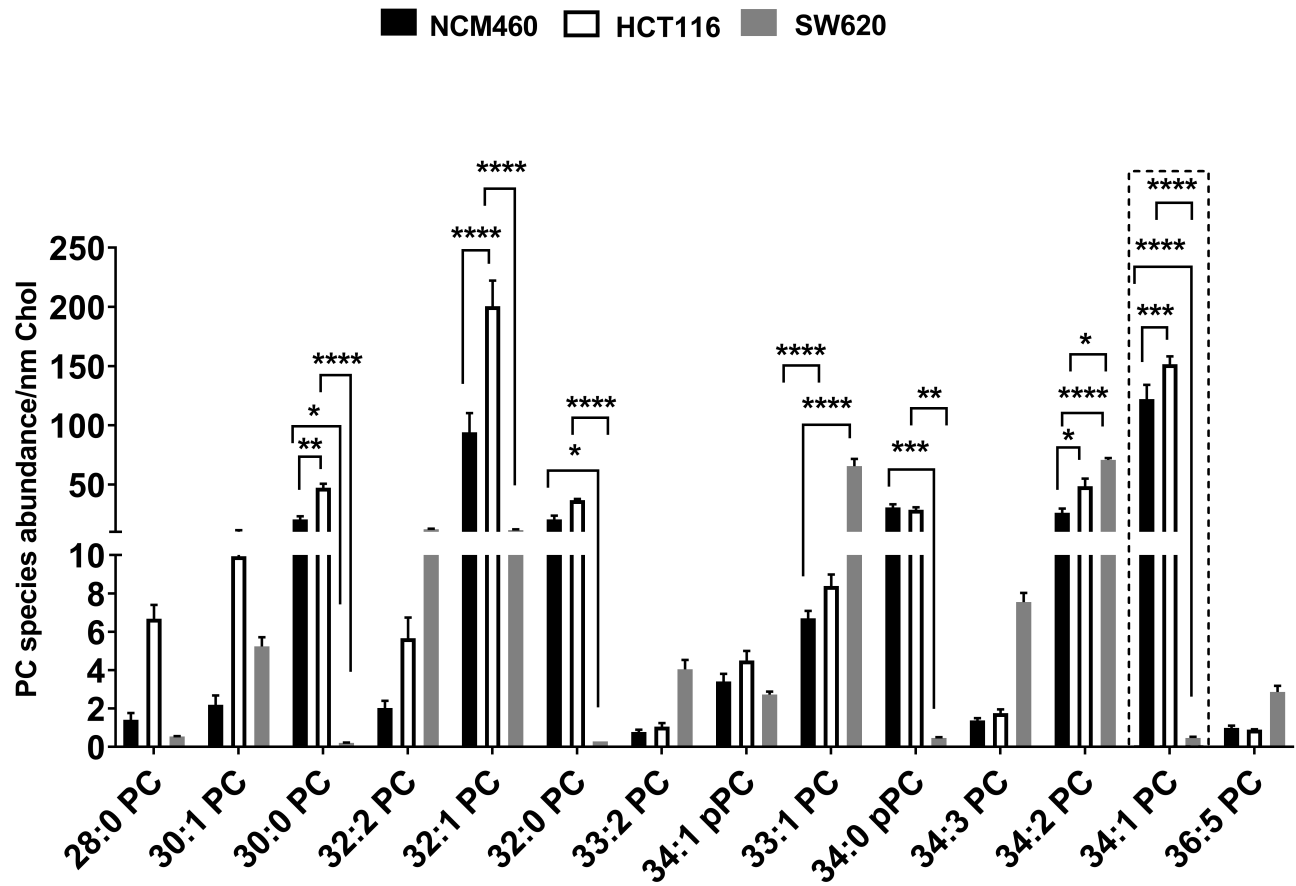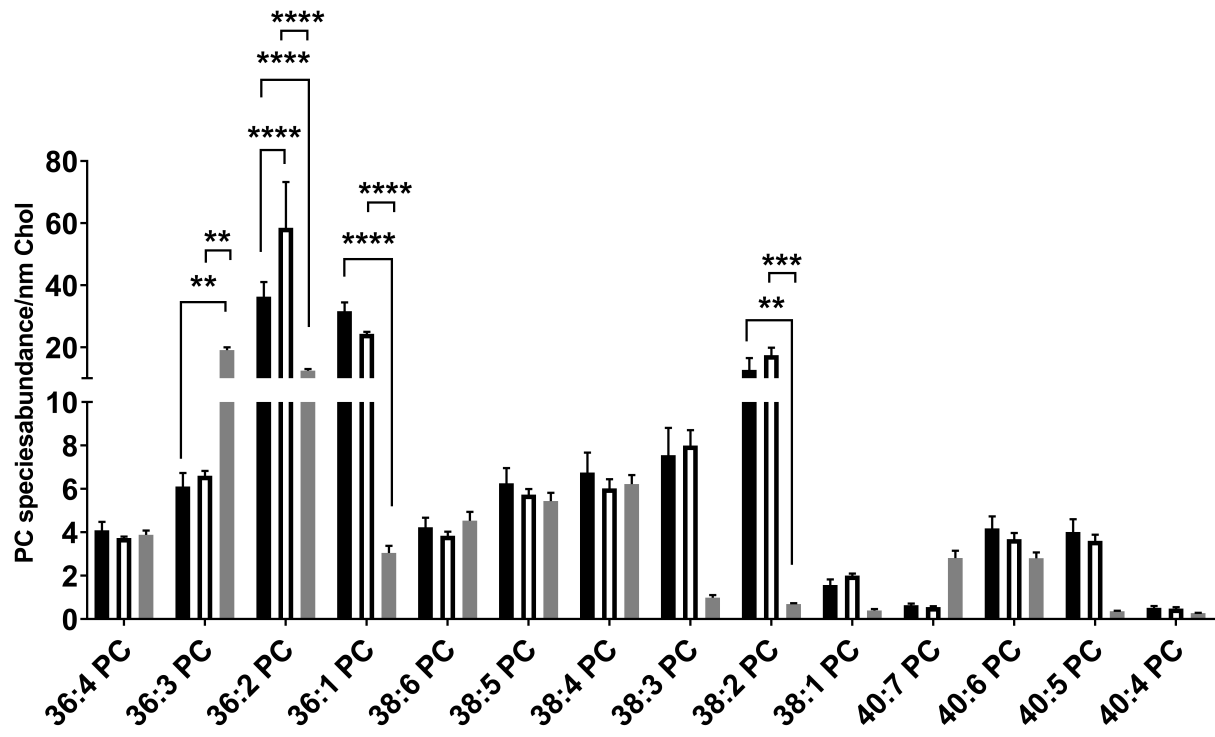

(B)      ■ Control      □ Patients (NM)      ■ Patients (M)

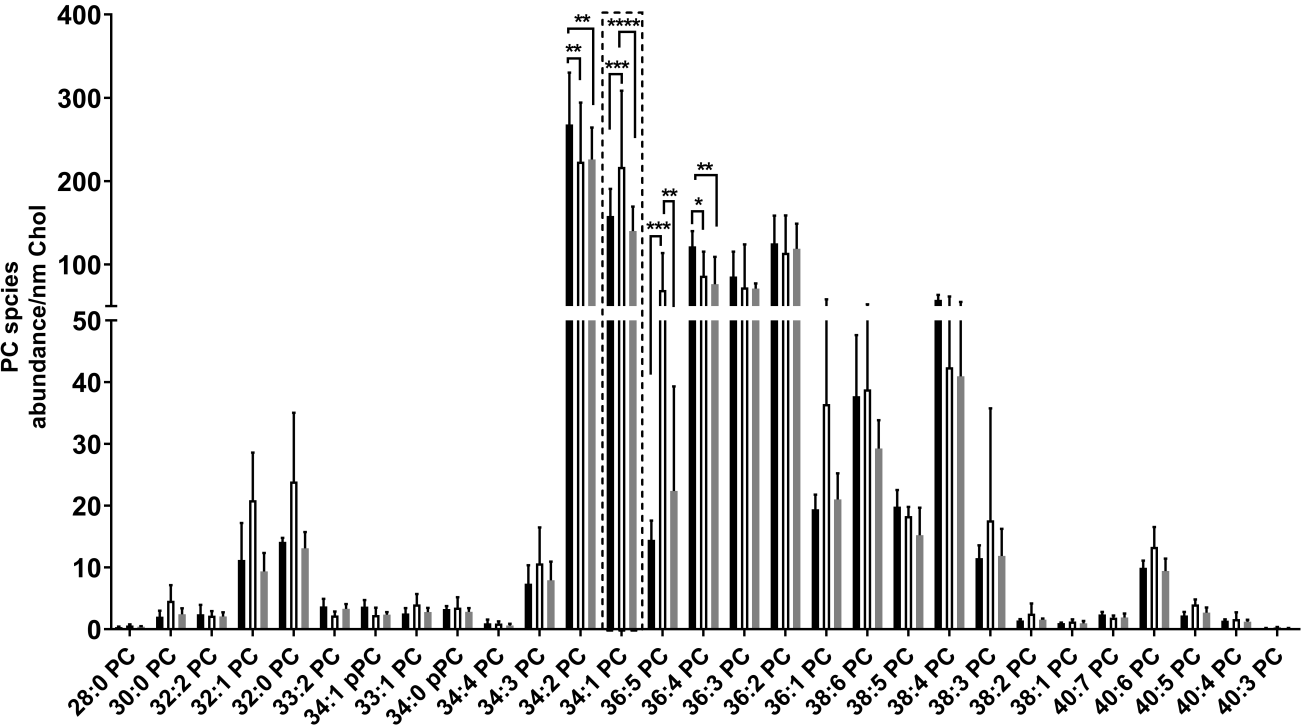

Supplement: Supplementary file 4 — Fig. S4. Phosphatidylcholine (PC) species analysis normalized to total cholesterol of exosomes derived from (A) normal colon mucosa NCM460D, nonmetastatic HCT116, and metastatic SW620 colorectal cancer (CRC) cell lines and from (B) Plasma‐derived exosomes of healthy donors and CRC patients (nonmetastatic and metastatic) illustrating an overall enrichment of 34:1 PC molecular species in both nonmetastatic cells and patients compared with their corresponding controls and metastatic counterparts. Data were analyzed by two‐way ANOVA followed by the Tukey’s multiple comparison test. Error bars represent standard error mean values (±SEM, n = 4). *P ≤ 0.05, **P ≤ 0.01, ***P ≤ 0.001, ****P ≤ 0.0001. [file MOL2-16-2710-s005.pdf]

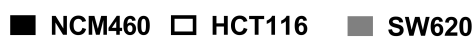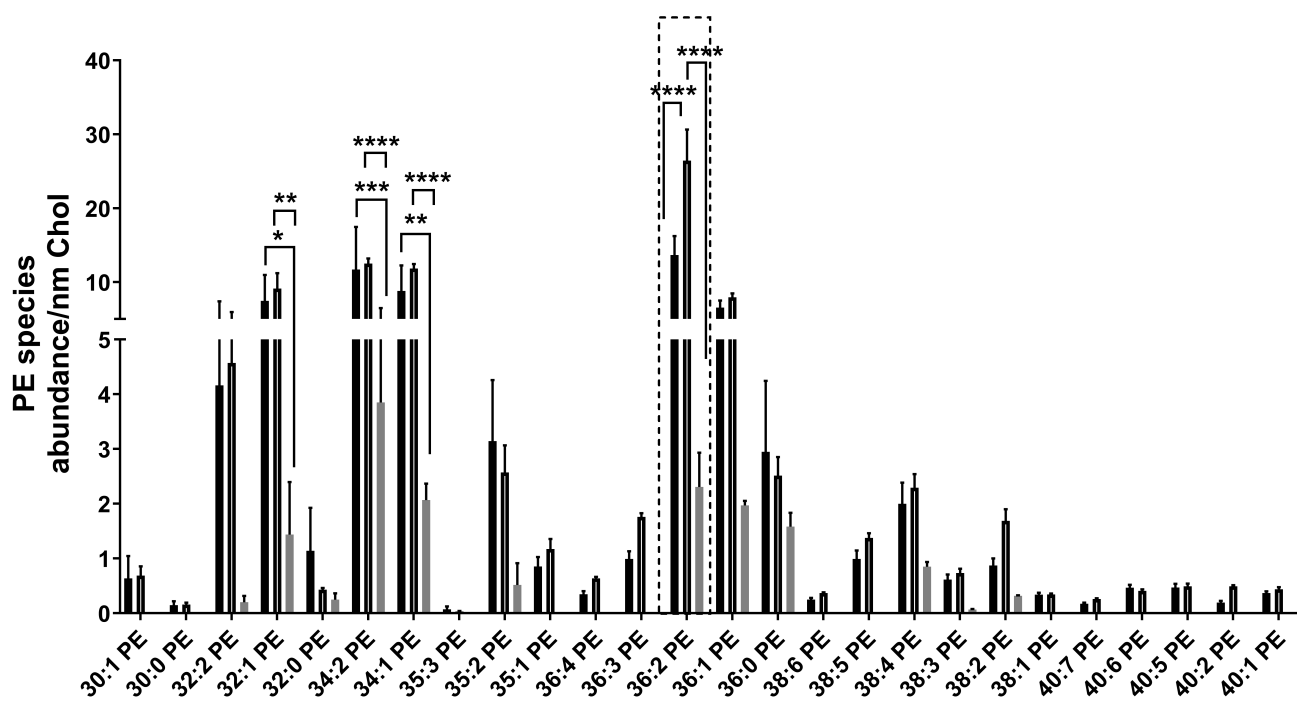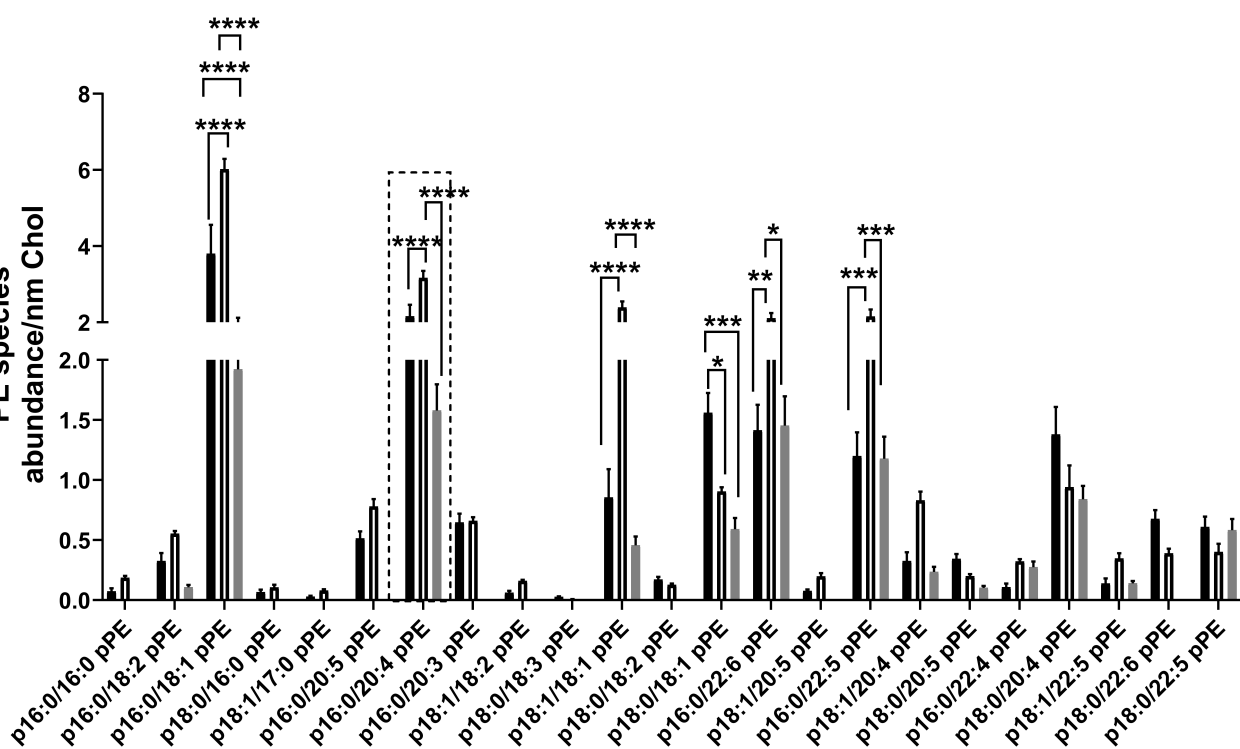

(B)

■ Control    □ Patients (NM)    ■ Patients (M)

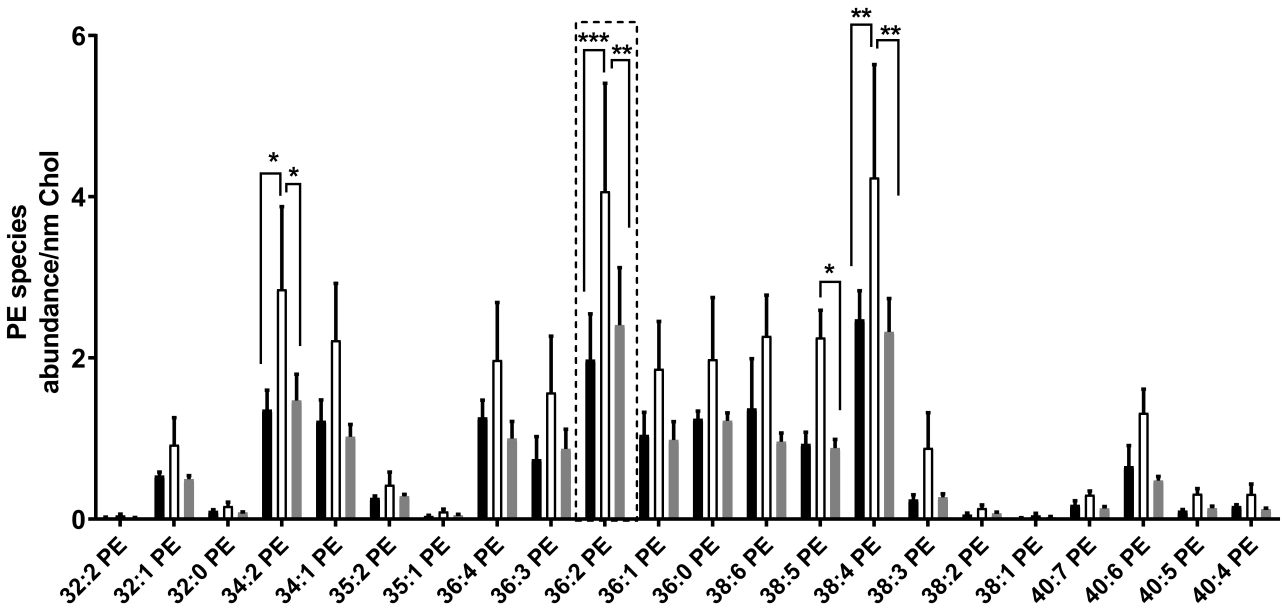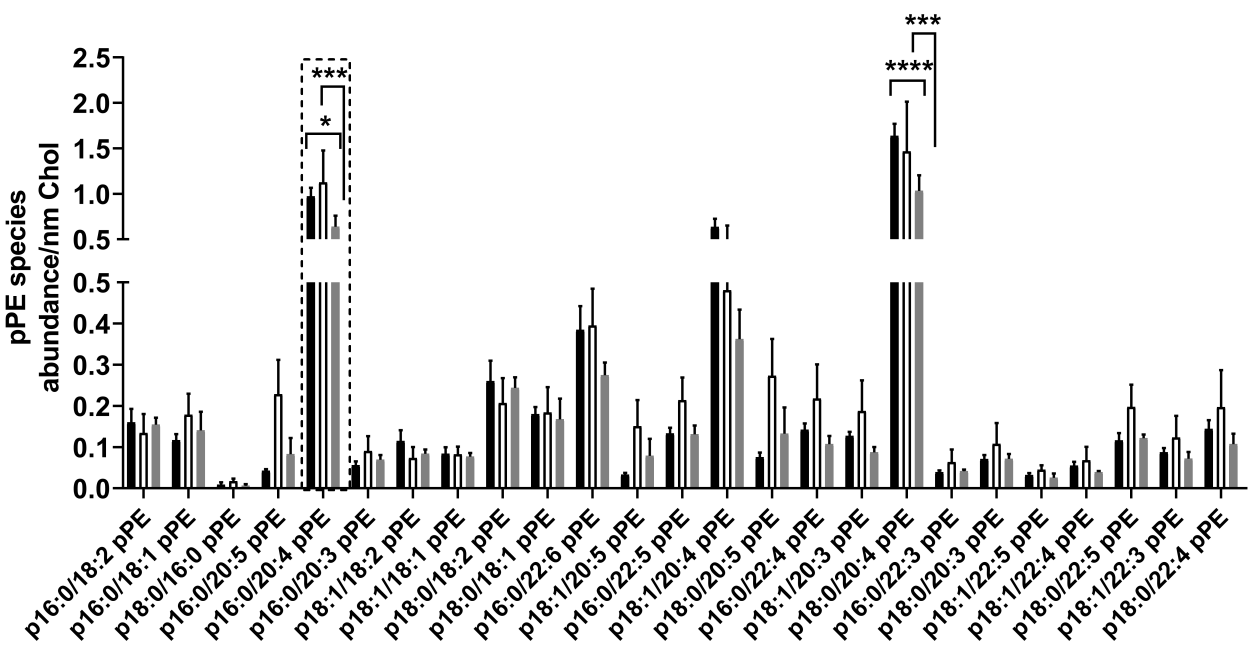

Supplement: Supplementary file 5 — Fig. S5. Phosphatidylethanolamine (PE) and plasmalogen (pPE) molecular species analysis normalized to total cholesterol in exosomes derived from (A) normal colon mucosa NCM460D, nonmetastatic HCT116 and metastatic SW620 colorectal cancer (CRC) cell lines and from (B) plasma‐derived exosomes of healthy donors and CRC patients nonmetastatic and metastatic (n = 4 for each group, pooled). Exosomes from both nonmetastatic HCT116 cells, and patients showed a significant increase of PE species 36:2 compared with their corresponding controls and metastatic counterparts. Metastatic SW620 cells and patients revealed a significant decrease in p16:0/20:4 pPE level compared with their nonmetastatic counterparts. Data were analyzed by two‐way ANOVA followed by the Tukey’s multiple comparison test. Error bars represent standard deviation (±SD, n = 4) values. *P ≤ 0.05, **P ≤ 0.01, ***P ≤ 0.001, ****P ≤ 0.0001. [file MOL2-16-2710-s001.pdf]

(A)

■ NCM460 □ HCT116 ■ SW620

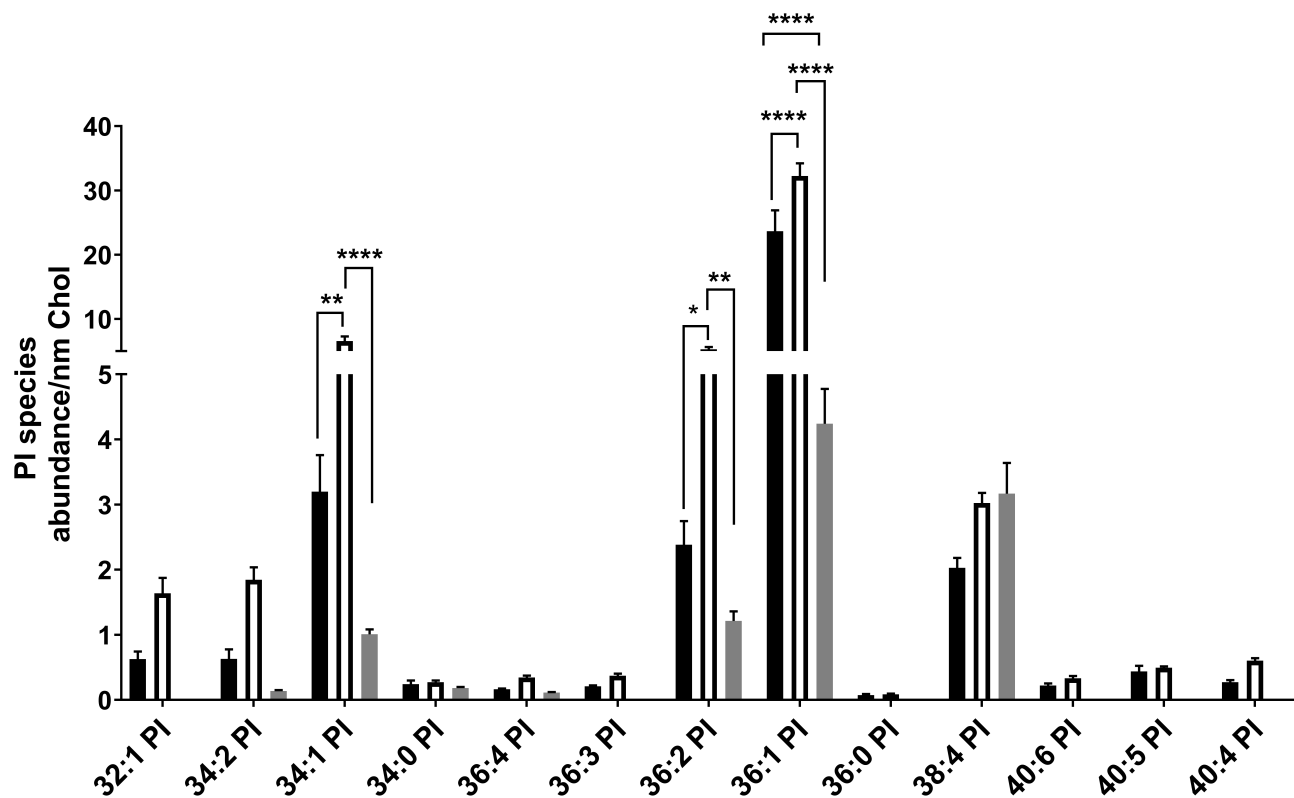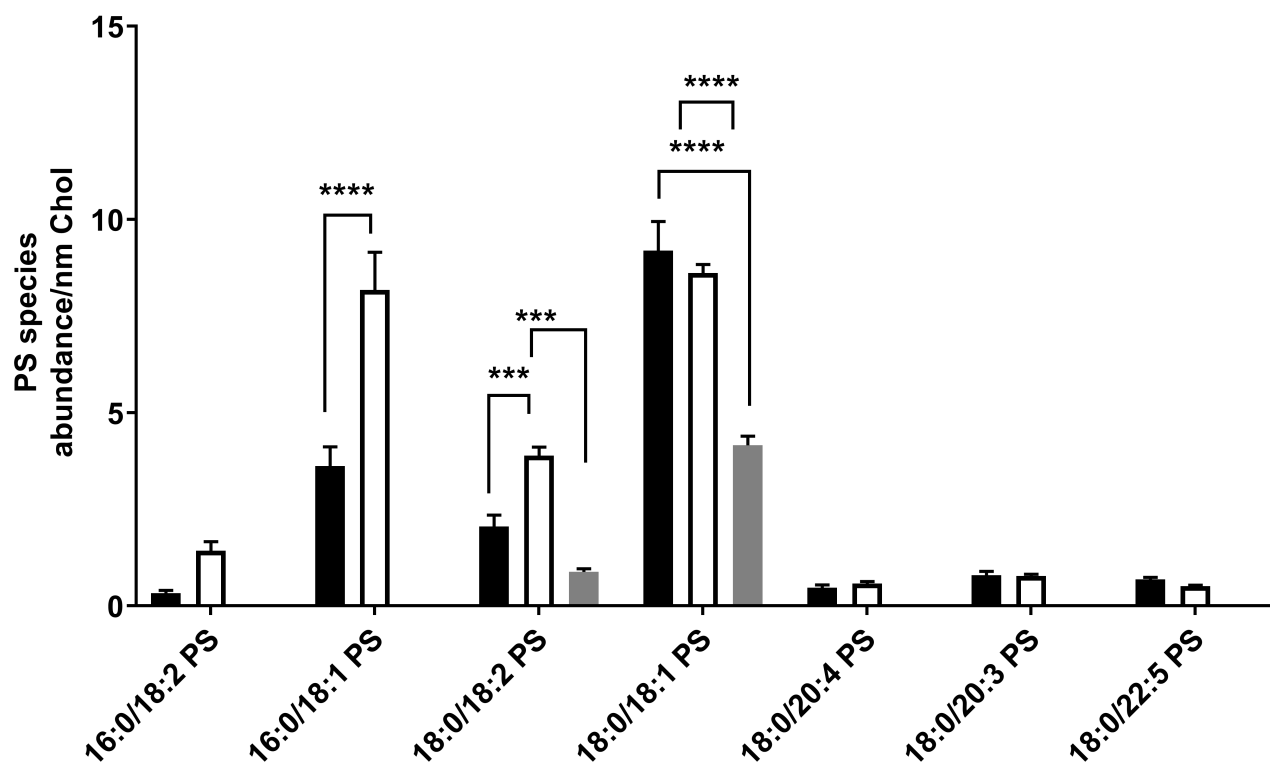

(B)

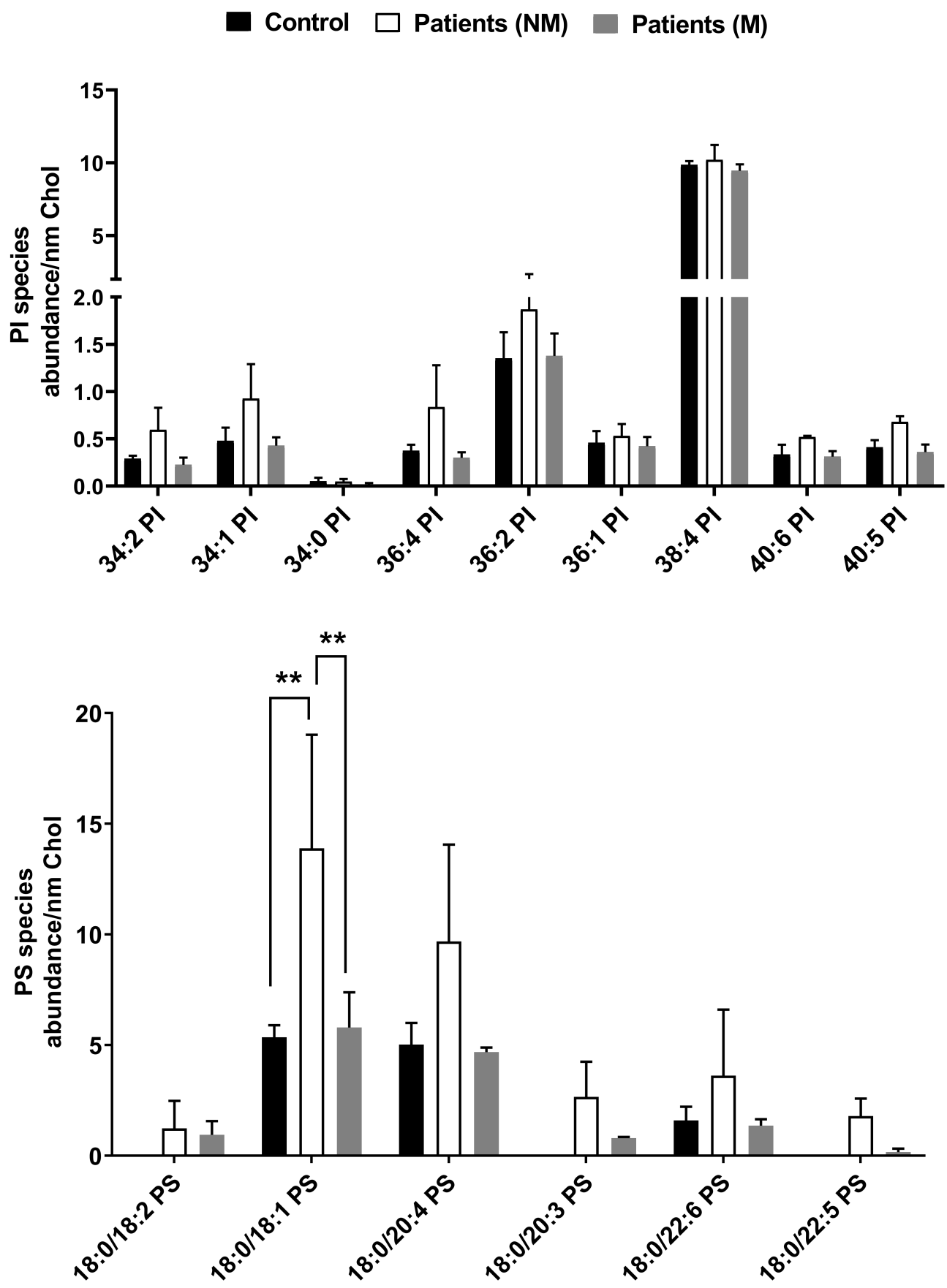

Supplement: Supplementary file 6 — Fig. S6. Phosphatidylinositol (PI) and phosphatidylserine (PS) molecular species analysis of exosomes derived from (A) normal colon mucosa NCM460D, nonmetastatic HCT116, and metastatic SW620 colorectal cancer (CRC) cell lines and from (B) plasma‐derived exosomes of healthy donors and CRC patients nonmetastatic and metastatic (n = 4 for each group, pooled) normalized to total cholesterol. HCT116‐derived exosomes are enriched in the PI molecular species PI 34:1, 36:2, and 36:1 compared with NCM460D and SW620. No significant change in the level of PI species was detected in all exosomes derived from the plasma of healthy donors and patients. Data were analyzed by two‐way ANOVA followed by the Tukey’s multiple comparison test. Error bars represent standard deviation (±SD, n = 4) values. *P ≤ 0.05, **P ≤ 0.01, ***P ≤ 0.001, ****P ≤ 0.0001. [file MOL2-16-2710-s009.pdf]

(A)

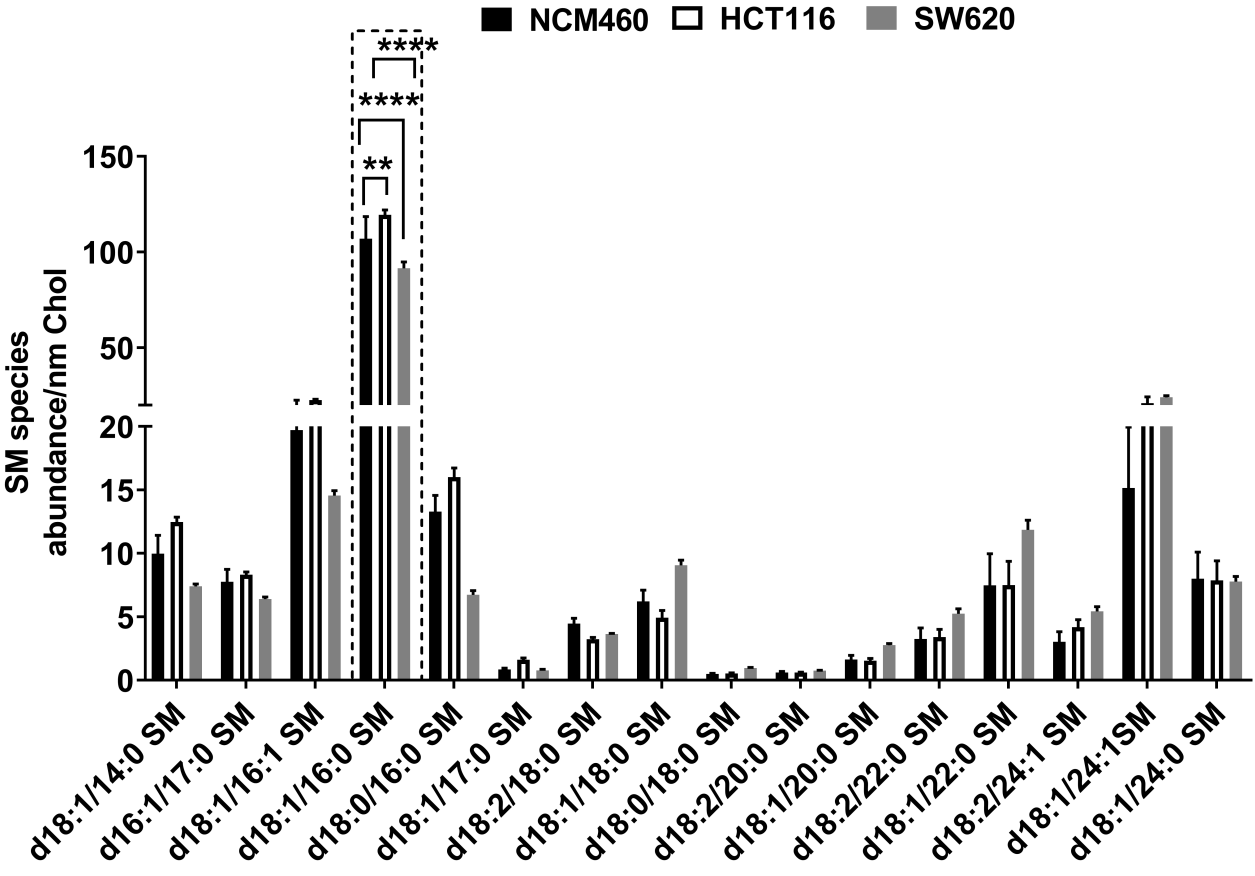

(B)

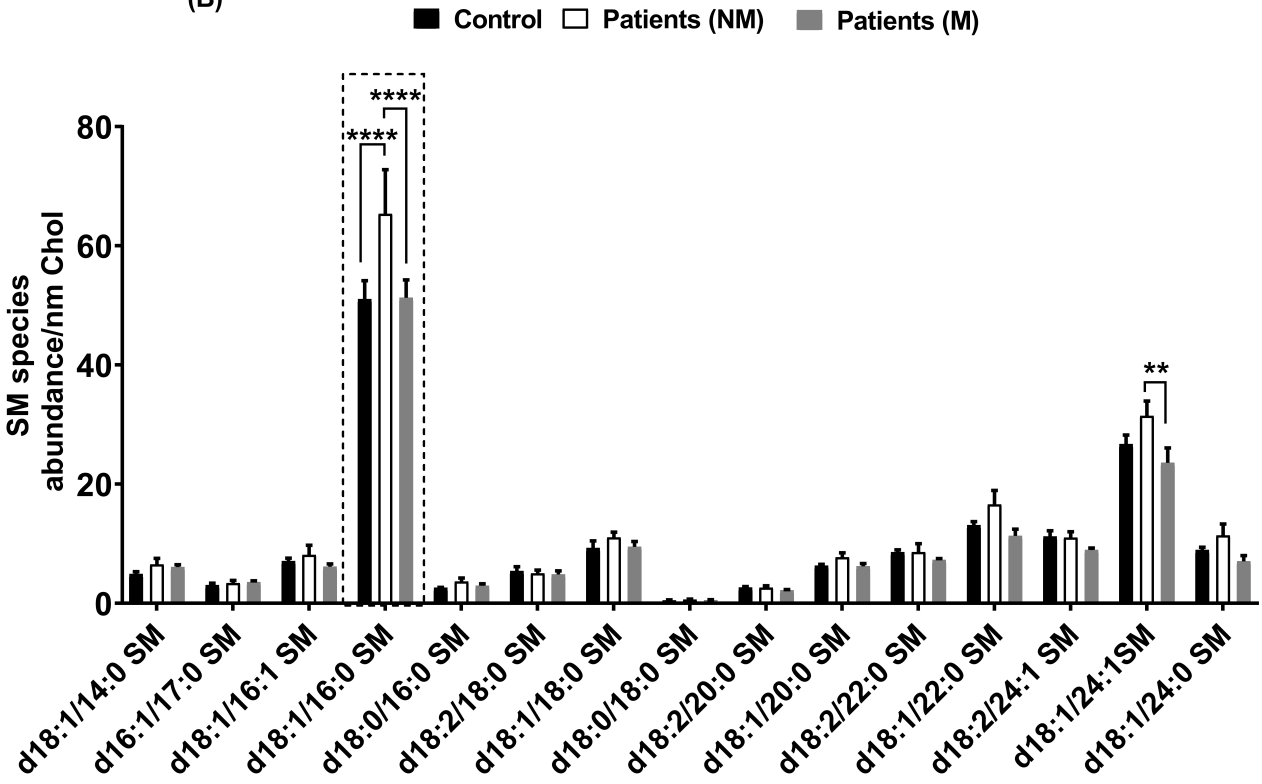

Supplement: Supplementary file 7 — Fig. S7. Analysis of sphingomyelin (SM) molecular species in exosomes derived from (A) normal colon mucosa NCM460D, nonmetastatic HCT116, and metastatic SW620 colorectal cancer (CRC) cell lines and from (B) plasma‐derived exosomes of healthy donors and CRC patients nonmetastatic and metastatic normalized to total cholesterol (n = 4 for each group, pooled). Both nonmetastatic HCT116‐ and patient‐derived exosomes revealed a marked increase in the level of d18:1/16:0 SM molecular species compared with their corresponding controls and metastatic counterparts. Data were analyzed by two‐way ANOVA followed by the Tukey’s multiple comparison test. Error bars represent standard deviation (±SD, n = 4) values. *P ≤ 0.05, **P ≤ 0.01, ***P ≤ 0.001, ****P ≤ 0.0001. [file MOL2-16-2710-s003.pdf]

(A)

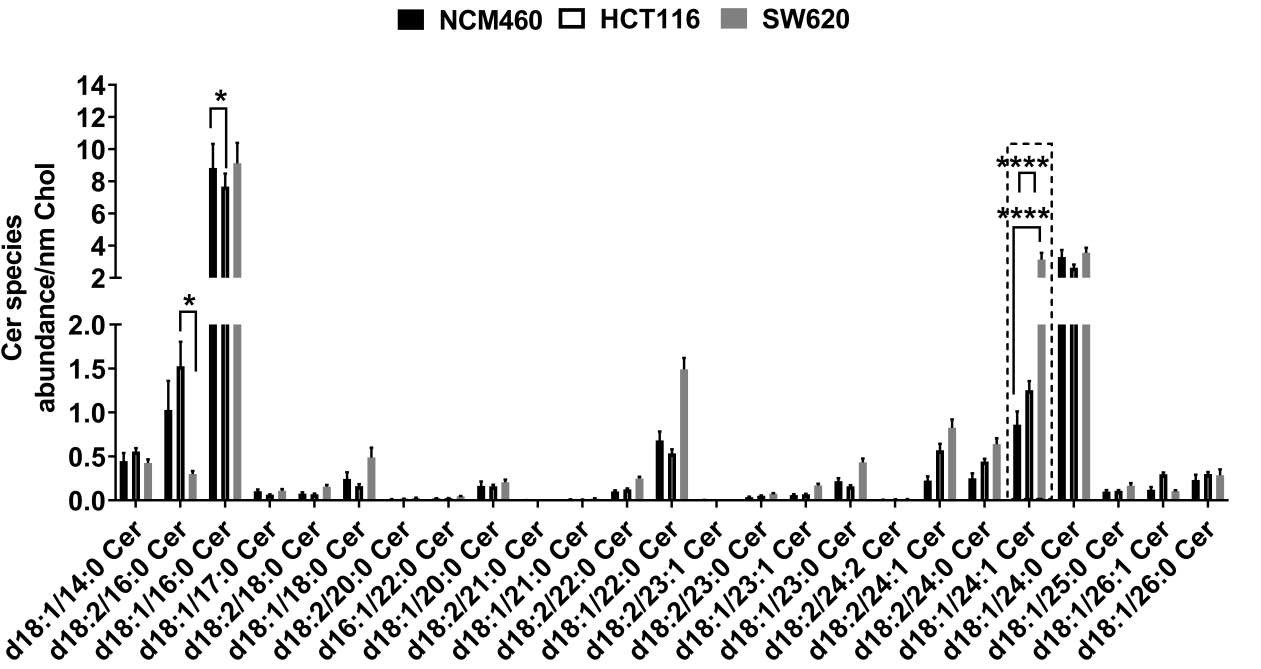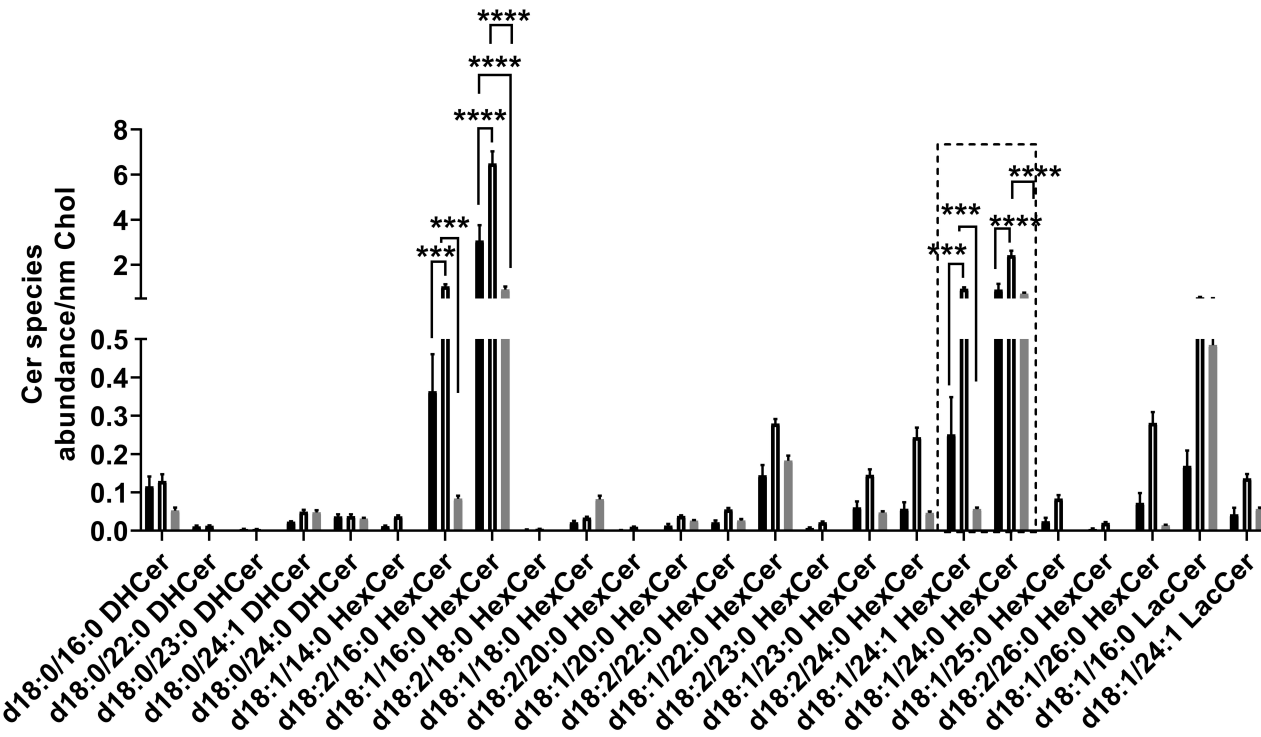

(B)

Control Patients (NM) Patients (M)

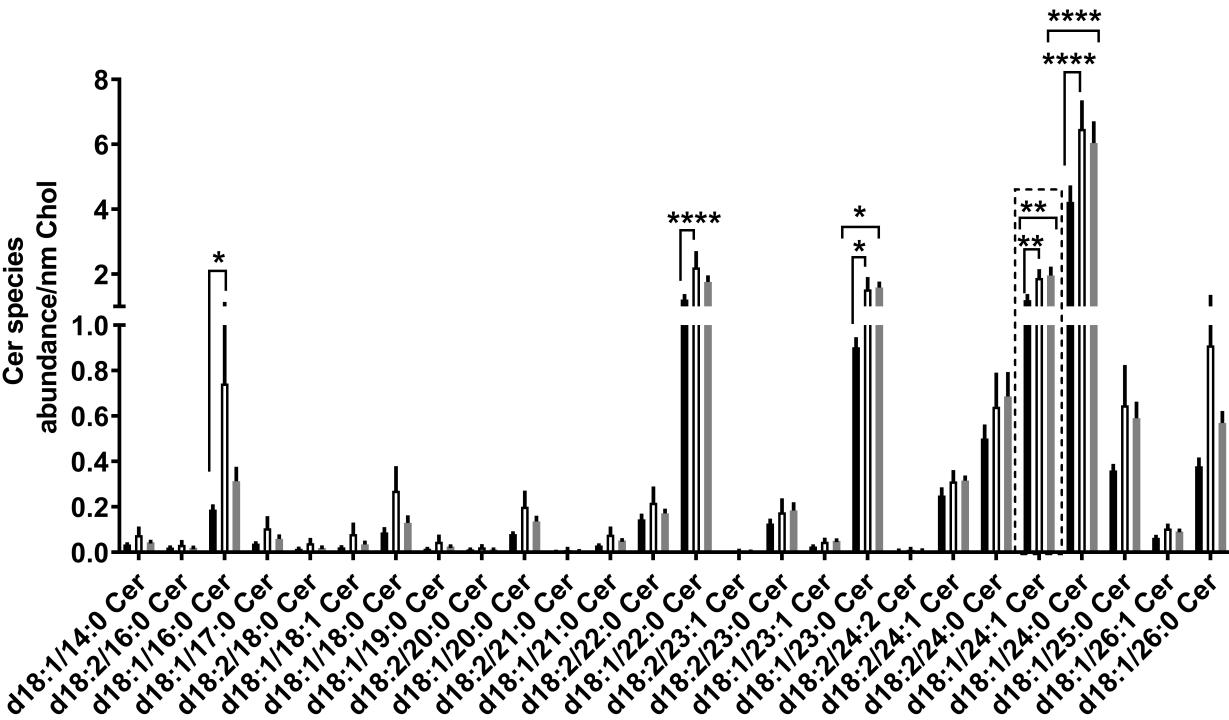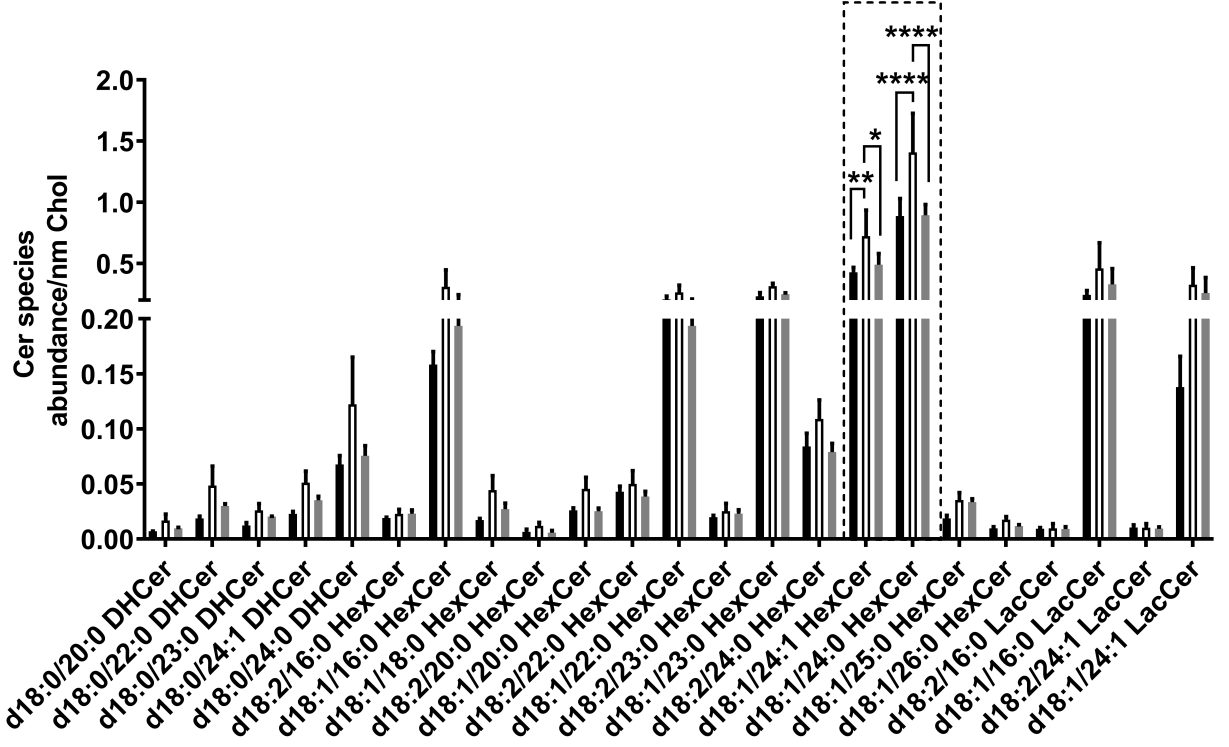

Supplement: Supplementary file 8 — Fig. S8. Ceramide (Cer) molecular species analysis of exosomes derived from (A) normal colon mucosa NCM460D, nonmetastatic HCT116, and metastatic SW620 colorectal cancer (CRC) cell lines and from (B) plasma‐derived exosomes of healthy donors and CRC patients nonmetastatic and metastatic, normalized to total cholesterol (n = 4 for each group, pooled). Nonmetastatic HCT116‐ and patient‐derived exosomes had an increase in the level of hexosylceramide d18:1/24:1 HexCer and d18:1/24:0 HexCer molecular species compared with their controls and metastatic counterparts. Both metastatic SW620‐ and patient‐derived exosomes displayed a significant increase in the ceramide molecular species d18:1/24:1 compared with their controls. Data were analyzed by two‐way ANOVA followed by the Tukey’s multiple comparison test. Error bars ±SD, n = 4. *P ≤ 0.05, **P ≤ 0.01, ***P ≤ 0.001, ****P ≤ 0.0001. [file MOL2-16-2710-s002.pdf]
