# Supplementary material for: Lipidomic profiling of exosomes from colorectal cancer cells and patients reveals potential biomarkers
Source: Mol Oncol. 2022 Jun 14;16(14):2710–8. doi: 10.1002/1878-0261.13223 (PMC9298677; doi:10.1002/1878-0261.13223)
Supplement: Supplementary file 9 — Table S1. Total lipid ions quantified by liquid chromatography–mass spectrometry (LC‐MS) in exosomes derived from normal colon mucosa NCM460D, nonmetastatic HCT116, and metastatic SW620 colorectal cancer (CRC) cell lines and from plasma‐derived exosomes of healthy controls (HC), and CRC patients nonmetastatic (NM) and metastatic (M). [file MOL2-16-2710-s007.pdf]

Table S1

Lipid Class	Lipid sub-class	Cell line-derived exosomes			Plasma-derived exosomes		
		NCM460	HCT116	SW620	Healthy control (HC)	Non-metastatic (NM) patients	Metastatic (M) patients
		Total lipid ion					
Phospholipids	LPC	7	7	5	13	13	13
	PC	28	28	27	29	29	29
	LPE	3	3	5	7	7	7
	PE	26	26	12	22	22	22
	pPE	23	23	17	25	25	25
	LPS	2	2	1	-	-	-
	PS	7	7	2	8	8	8
	PI	13	13	7	9	9	9
Sphingolipids	SM	16	16	16	15	15	15
	Cer	25	25	23	26	26	26
	HexCer	17	17	11	14	14	14
	LacCer	2	2	2	4	4	4
	DHCer	5	5	3	5	5	5
Sterol lipids	Chol	1	1	1	1	1	1
Total lipid		175	175	132	178	178	178
